# Supplementary material for: Functional diversity of soil microbial communities increases with ecosystem development
Source: Nat Commun. 2025 Nov 22;16:10408. doi: 10.1038/s41467-025-66544-8 (PMC12644890; doi:10.1038/s41467-025-66544-8)
Supplement: Supplementary file 1 — Supplementary Information [file 41467_2025_66544_MOESM1_ESM.pdf]

SUPPLEMENTARY MATERIALS

Accompanying the article

Functional diversity of soil microbial communities increases with ecosystem development

SUPPLEMENTARY FIGURES

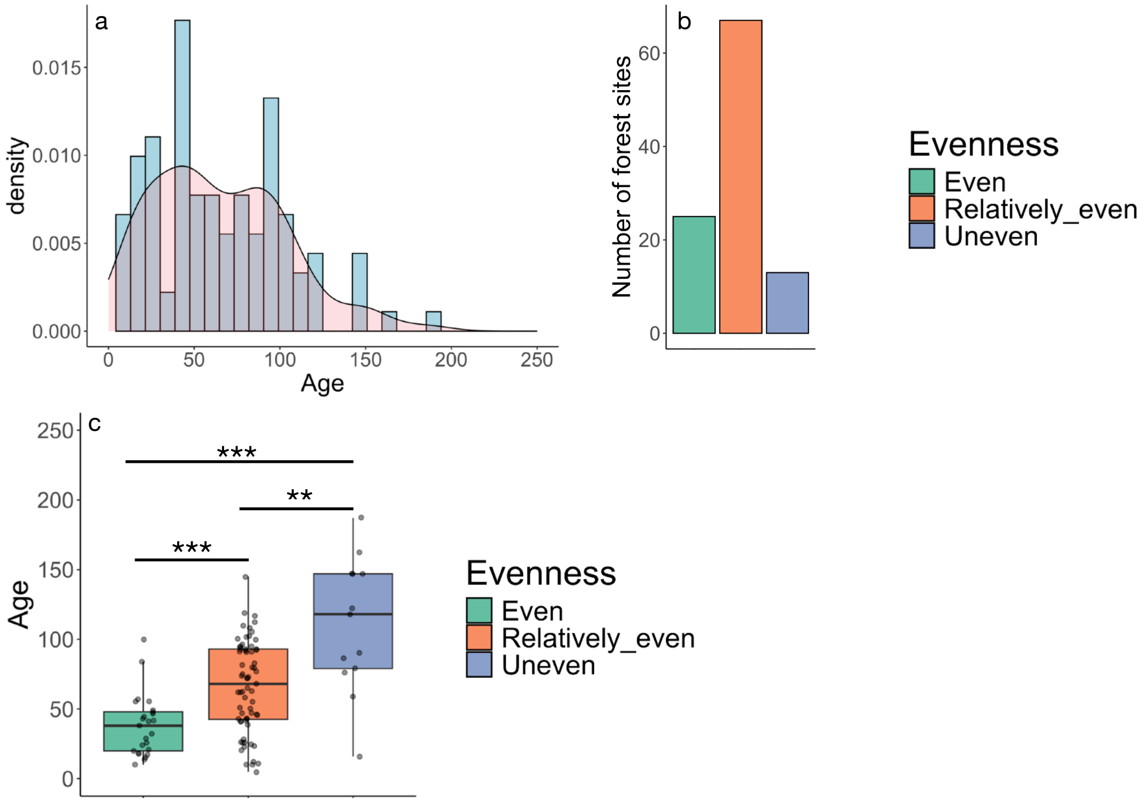

Fig. S1: Structure of the forest sites used as successional endpoints in the study
Density plot (**a**) showing the the proportion of forest sites across differing age classes. In panel (**b**) the same forest sites are shown distributed according to the stand evenness. Boxplot (**c**) showing differences in stand age among the different evenness classes, with asterisks (*) denoting significant differences based on pairwise Wilcoxon tests with the following significance levels: *p 0.05, **p 0.01, ***p 0.001. Source data and codes to reproduce this figure are provided at https://zenodo.org/records/17176048.

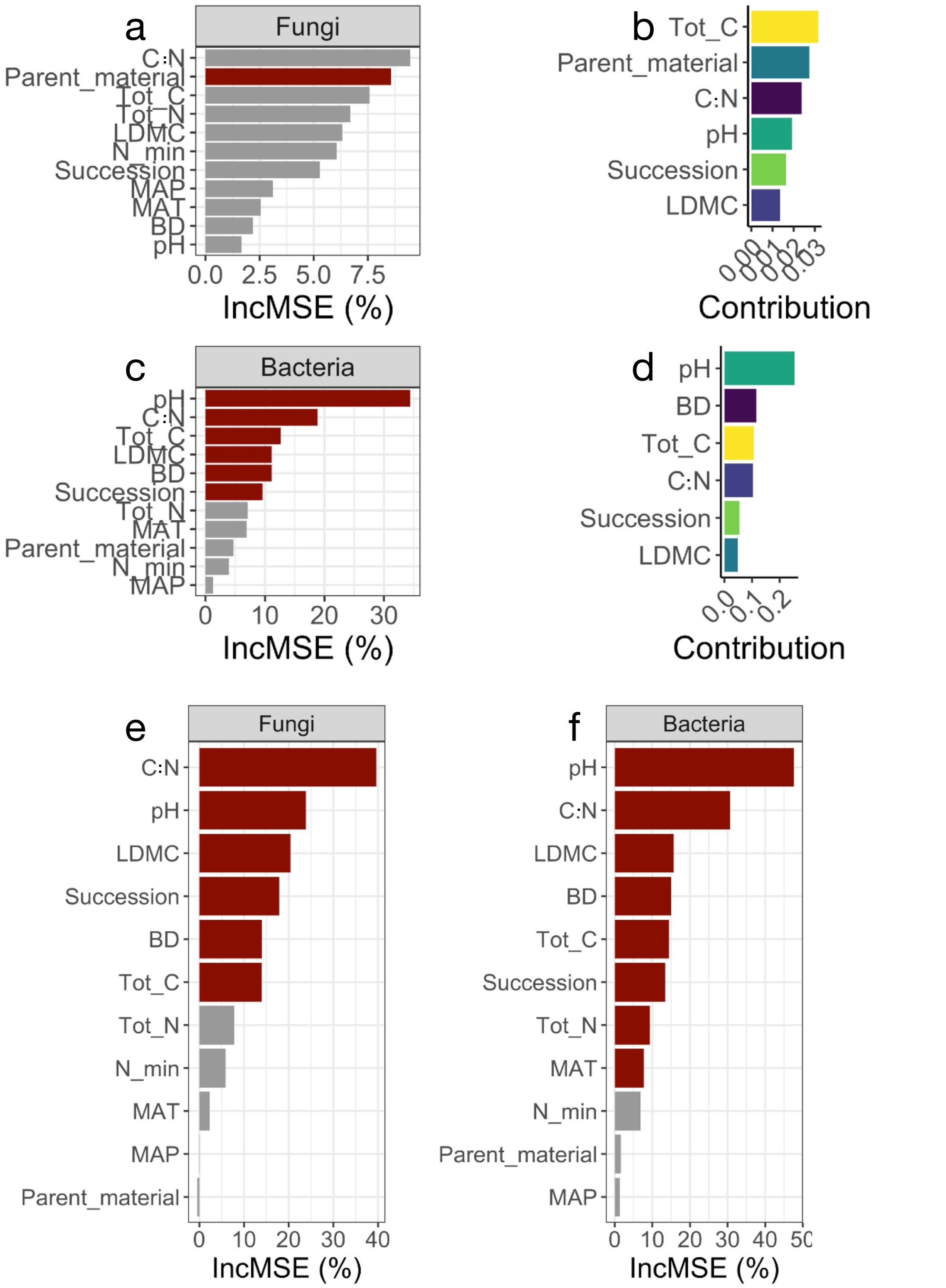

Fig. S2: Abiotic drivers of microbial taxonomic diversity and their relative contributions
Results from permutation-based random-forest variable selection showing the importance of environmental factors in explaining (**a**) fungal taxonomic diversity, **(c**) bacterial taxonomic diversity, as well as (**e**) fungal and (**f**) bacterial community composition. IncMSE (%) describes the relative importance of the explanatory variable in the predicted response variable. Red color denotes that the explanatory variable is significantly (*p* 0.05) linked to the response variable.
The relative contribution of the main factors identified through variable selection were also partitioned using hierarchical partitioning analyses for (**b**) fungal, and (**d**) bacterial diversity, respectively. BD = bulk density, MAT = mean annual temperature, MAP = mean annual precipitation, LDMC = leaf dry matter content, N_min = mineral Nitrogen. Source data and codes to reproduce this figure are provided at https://zenodo.org/records/17176048.

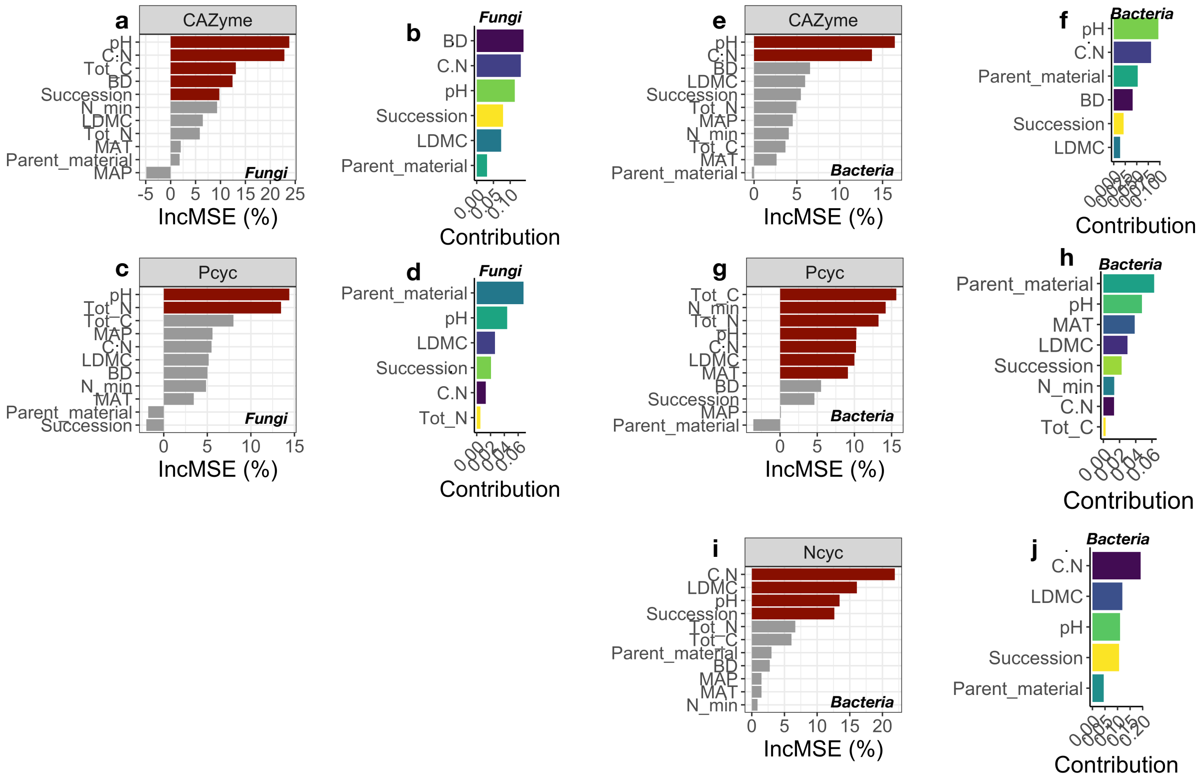

Fig. S3: Abiotic drivers of microbial functional diversity and their relative contributions
Results from permutation-based random-forest variable selection showing the importance of environmental factors in explaining fungal (**a**) C-cycling and (**c**) P-cycling genetic diversity, together with their relative contributions (**b,d**). Panels (**e,g,i**) show corresponding results for bacterial C-P-N cycling gene diversity, together with their respective hierarchical partitioning (**f,h,j**). IncMSE (%) describes the relative importance of the explanatory variable in the predicted response variable. Red color denotes that the explanatory variable is significantly (p 0.05) linked to the response variable.
BD = bulk density, MAT = mean annual temperature, MAP = mean annual precipitation, LDMC = leaf dry matter content, N_min = mineral Nitrogen. Source data and codes to reproduce this figure are provided at https://zenodo.org/records/17176048.

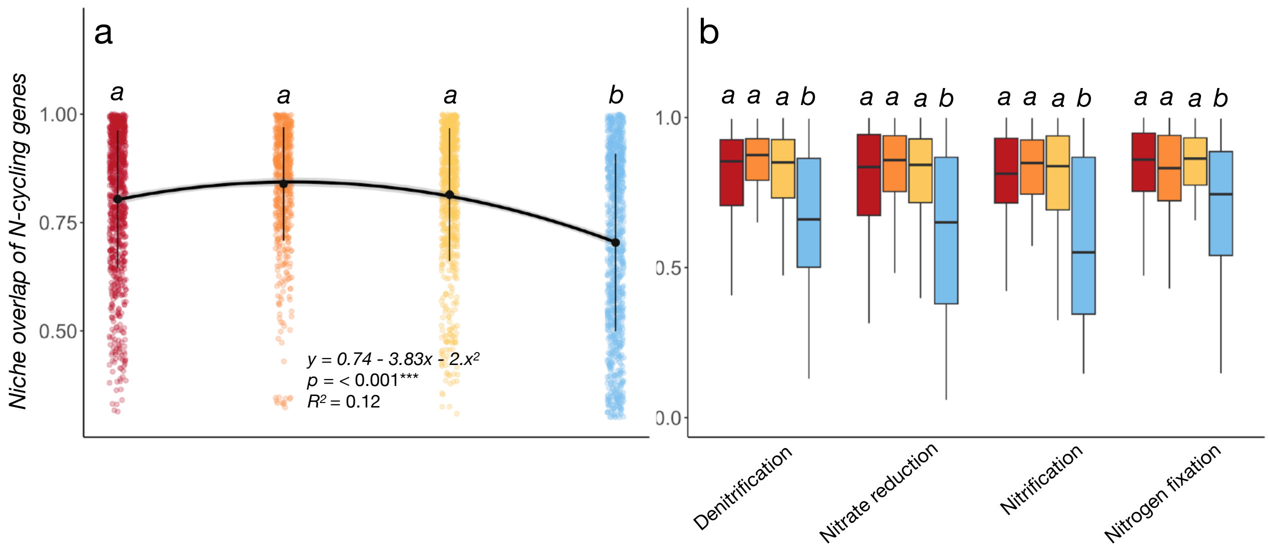

Fig. S4: Genetic overlap of N-cycling genes and pathways

Genetic overlap of **a**) bacterial N-cycling genes across the land abandonment gradient. Black points show mean genetic overlap ± s.d. (vertical lines) for each land-use stage. Horizontal lines connecting land-use stages indicate significant (*p* 0.05) ordinary least-square or second-order polynomial regression fits (Table S12). In panel **b**) the overlaps are partitioned across key N-cycling pathways. Letters indicate significant differences (*p* 0.05) between land use-stages based on pairwise Wilcoxon Rank-Sum Tests, with full test result details found in Table S15 & Table S18. Source data and codes to reproduce this figure are provided at https://zenodo.org/records/17176048.

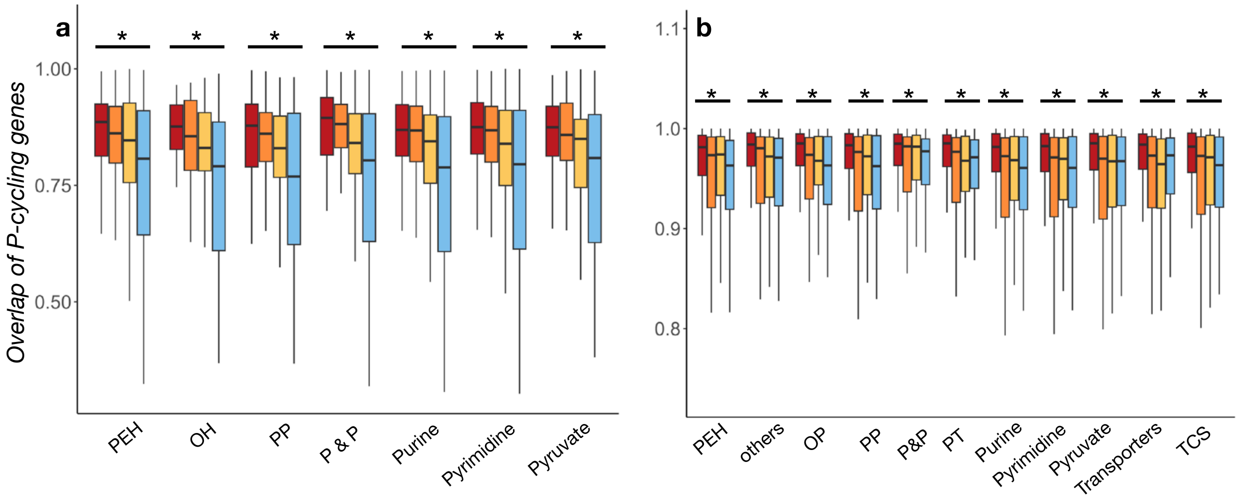

Fig. S5: Genetic overlap of P-cycling pathways

Genetic overlap of **a**) fungal and **b**) bacterial P-cycling genes partitioned across annotated pathways. Stars (*) above these pathways indicate significant (*p* 0.05) differences between one or more land-use stages based on pairwise Wilcoxon tests, with full test result details found in Table S17.

PEH = Phosphoester hydrolysis; OH = Organic phosphoester hydrolysis; PP = Pentose phosphate; P&P = Phosphonate and Phospinate metabolism; OP = Oxidative phosphorylation; PT = Phosphotransferase; TCS = Two component system.

Source data and codes to reproduce this figure are provided at https://zenodo.org/records/17176048**.**

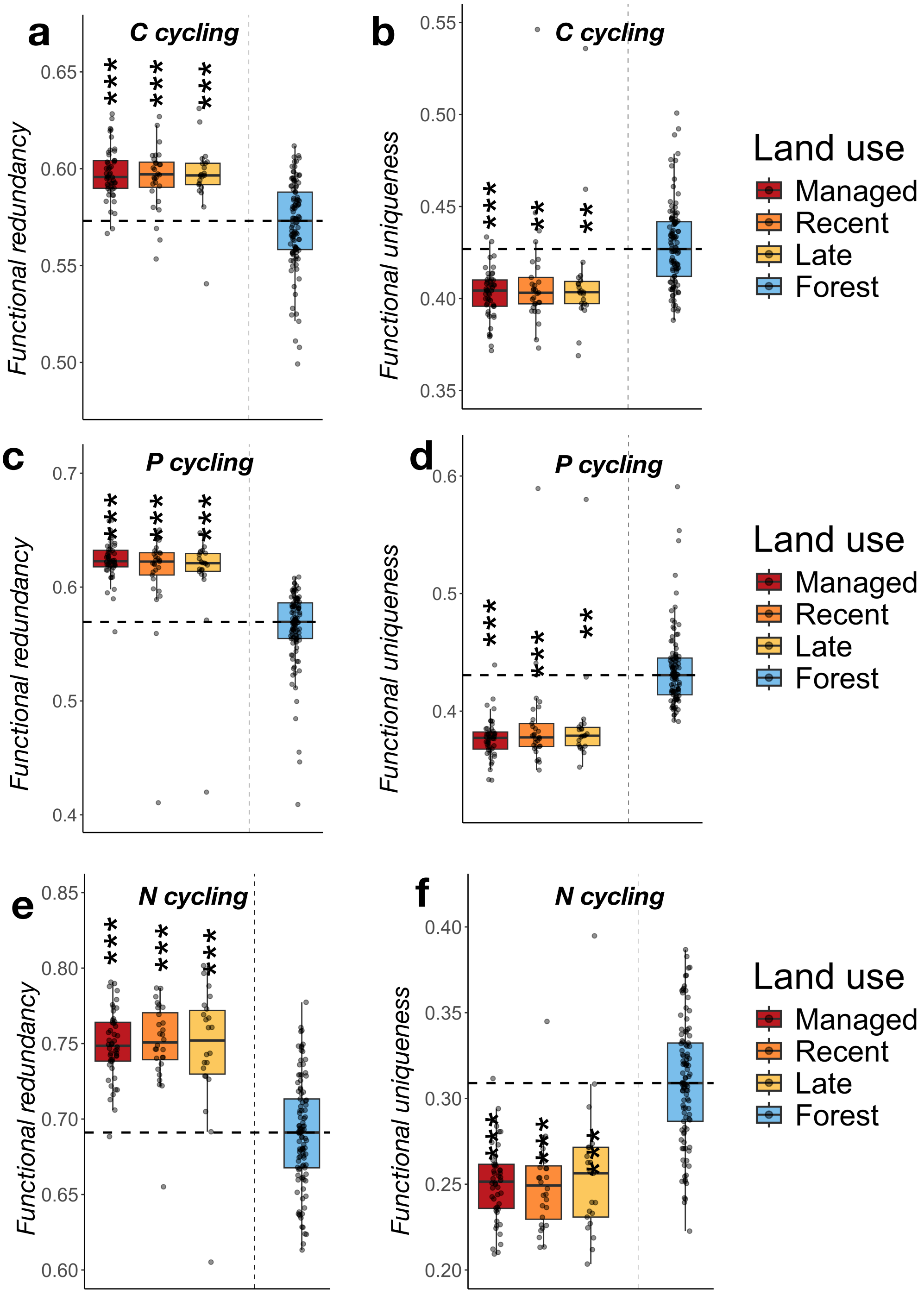

Fig. S6: Functional redundancy and specialization

Boxplots showing levels of functional redundancy and functional uniqueness (specialization) across **a**) C-cycling, **b**) P-cycling, and **c**) N-cycling genes for bacterial communities based on predicted metagenomes.

The lower and upper hinges of the boxplots represent the 25th and 75th percentiles, respectively, and the middle line is the median. The whiskers extend from the median by 1.5x the interquartile range. The dashed horizontal line indicates the median of the forest sites, with asterisks (*) denoting significant differences between paired grassland and forest sites based on mixed-effect linear models accounting for paired structure and spatial distance with the following significance levels: **p* 0.05, ***p* 0.01, ****p* 0.001. Source data and codes to reproduce this figure are provided at https://zenodo.org/records/17176048.

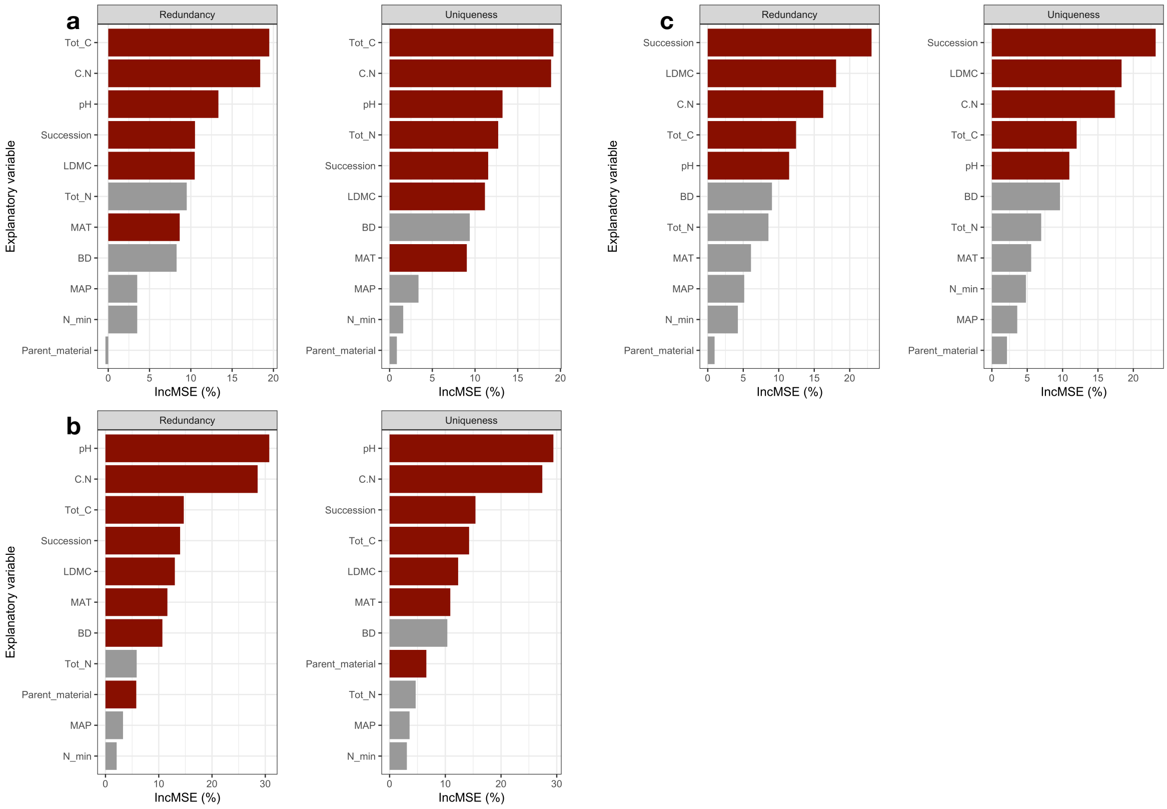

Fig. S7: Drivers of functional redundancy and specialization
Barplots showing results from random-forest based variable selection for drivers of functional redundancy and functional uniqueness (i.e. specialization) across **a**) C-cycling, **b**) P-cycling, and **c**) N-cycling genes for bacterial communities based on predicted metagenomes. IncMSE (%) describes the relative importance of the explanatory variable in the predicted response variable. Red color denotes that the explanatory variable is significantly (*p* 0.05) linked to the response variable.
BD = bulk density, MAT = mean annual temperature, MAP = mean annual precipitation, LDMC = leaf dry matter content, N_min = mineral Nitrogen. Source data and codes to reproduce this figure are provided at https://zenodo.org/records/17176048.

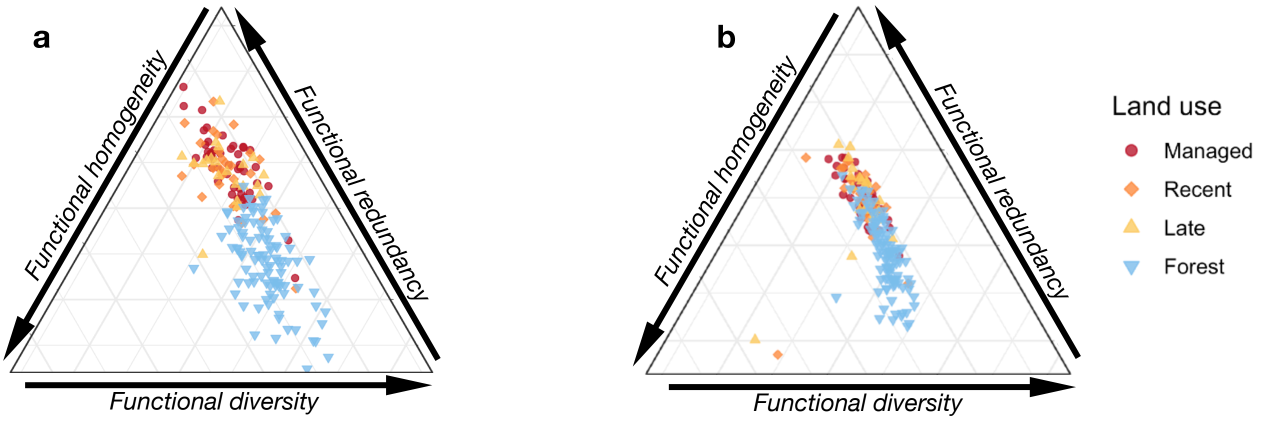

Fig. S8: Ternary plots of N- and P-cycling across the redundancy-specialization axis
Ternary plots showing the clustering of sites according to their placement along the successional gradient for **a**) C-cycling, **b**) P-cycling, and **c**) N-cycling genes. Source data and codes to reproduce this figure are provided at https://zenodo.org/records/17176048.

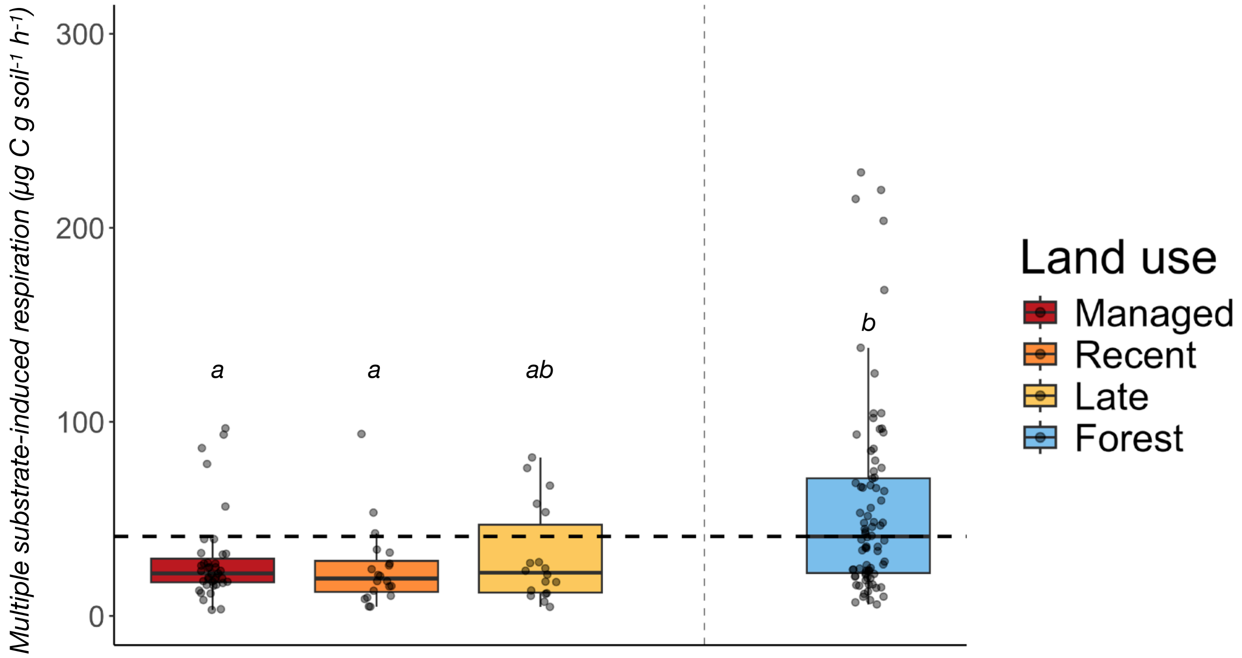

Fig. S9: Substrate degradation increases with afforestation
Aggregate values of multiple substrate-induced respiration (MSIR) from six differing substrates of differing complexity and recalcitrance across the land-use gradient. Letters denote significant (p 0.05) differences based on pairwise Wilcoxon tests. $N = 156$. Source data and codes to reproduce this figure are provided at https://zenodo.org/records/17176048.

SUPPLEMENTARY TABLES

Table S1: Site distribution for each dataset and land-use category across the successional gradient

**Dataset**	***Managed***	***Recent***	***Late-term***	***Forest***	***Sum***	***%Paired***
Metadata	49	30	23	105	207	92
Sequencing						
16S (amplicon)	48	29	22	105	204	90
ITS (amplicon)	47	26	22	104	199	88
Shotgun (metagenomes)	16	18	14	47	95	99
Experiment						
SIR	39	20	18	77	154	100

Table S2: Combined tests for differences in composition and indicator species contributing to observed differences between pairs of plant communities. All indicator species were significant (two-tailed MANOVA tests, p 0.05) with p-values adjusted for multiple comparisons (Benjamin-Hochberg)

**Comparison**	*SES*	*p-value*	*Indicator species*
Managed vs Recent	1.20	0.0072	*Taraxacum; T. repens; P. tremula*
Managed vs Late	2.59	0.0001	*R. arcticus; R. ideaus; F. ulmaria; C. purpurea; C. fontanum; Taraxacum; A millefolium; E. sylvaticum; P. lanceolata; L. vulgaris ; T. repens ; P. tremula ; B. pendula ; P. abies*
Recent vs Late	1.53	0.0012	*C. purpurea; A. millefolium; P. abies*

Table S3: Soil and leaf properties (mean ± SD) across the successional gradient. Letters indicate significant differences ($p < 0.05$). See Table S4 for exact test details

**Variable**	*Managed (n = 49)*	*Early (n = 30)*	*Late (n = 23)*	*Forest (n = 105)*
Soil properties				
pH	5.38 (0.41)^{a}	5.28 (0.28)^{a}	5.29 (0.52)^{a}	4.56 (0.54)^{b}
Total C (%)	9.25 (10.4)^{a}	9.06 (10.5)^{a}	11.0 (10.5)^{a}	17.9 (13.3)^{b}
Total N (%)	0.60 (0.60)	0.65 (0.73)	0.74 (0.64)	0.70 (0.52)
C:N	14.4 (2.87)^{a}	13.9 (2.96)^{a}	14.4 (2.22)^{a}	25.3 (6.66)^{b}
Available K (mg kg^{-1})	19.1 (16.7)	19.7 (13.0)	17.1 (6.37)	22.3 (13.1)
Available P (mg kg^{-1})	6.82 (6.15)	5.45 (3.29)	7.54 (8.66)	5.55 (3.12)
Available Fe (mg kg^{-1})	50.3 (43.3)^{a}	71.3 (86.7)^{ab}	100 (97.3)^{b}	45.7 (35.1)^{a}
Available Mg (mg kg^{-1})	15.7 (10.7)	17.5 (13.0)	16.9 (12.2)	18.2 (12.0)
Available Al (mg kg^{-1})	70.0 (60.7)	56.1 (32.8)	81.0 (47.0)	61.1 (47.2)
Leaf dry matter content (LDMC)	0.27 (0.04)^{a}	0.28 (0.04)^{a}	0.31 (0.04)^{b}	0.45 (0.02)^{c}

Table S4: Results from pairwise two-tailed Wilcoxon tests of differences in ecosystem properties across successional stages, with *p*-values corrected for multiple comparisons using Benjamin-Hochberg corrections.

Ecosystem property	***Contrast***	***n1***	***n2***	***statistic***	***p.adj***
pH	Managed vs Recent	49	30	891.5	0.339
pH	Managed vs Late	23	49	458	0.406
pH	Managed vs Forest	49	104	635	0.001***
pH	Recent vs Late	23	30	338.5	0.914
pH	Recent vs Forest	30	104	387.5	0.001***
pH	Late vs Forest	23	104	365	0.001***
Total C (%)	Managed vs Recent	49	30	809.5	0.455
Total C (%)	Managed vs Late	23	49	690.5	0.254
Total C (%)	Managed vs Forest	49	105	3877.5	0.001***
Total C (%)	Recent vs Late	23	30	463	0.103
Total C (%)	Recent vs Forest	30	105	2438.5	0.001***
Total C (%)	Late vs Forest	23	105	1662	0.019*
C:N	Managed vs Recent	49	30	782	1
C:N	Managed vs Late	23	49	592	1
C:N	Managed vs Forest	49	105	4846	0.001***
C:N	Recent vs Late	23	30	383.5	1
C:N	Recent vs Forest	30	105	3005	0.001***
C:N	Late vs Forest	23	105	2293.5	0.001***
Fe	Managed vs Recent	49	30	679.5	1
Fe	Managed vs Late	23	49	767	0.071
Fe	Managed vs Forest	49	104	2535	
Fe	Recent vs Late	23	30	444	0.308
Fe	Recent vs Forest	30	104	1447	1
Fe	Late vs Forest	23	104	742	0.027*
LDMC	Managed vs Recent	47	28	721	0.496
LDMC	Managed vs Late	23	47	686	0.139
LDMC	Managed vs Forest	47	101	4643	0.001***
LDMC	Recent vs Late	23	28	432	0.112
LDMC	Recent vs Forest	28	101	2827	0.001***
LDMC	Late vs Forest	23	101	2294	0.001***

Table S5: Number of reads and annotated orthologous groups (OG) related to nutrient cycling pathways obtained from the shotgun metagenomic sequencing of n = 95 samples across the successional gradient.

**Dataset**	***Reads***	***mean ± SD***	***OG***	
Total metagenome reads	3532698106	37186296 ± 7365270		
CAZyme fungi	637506	6710 ± 3720	236	
CAZyme bacteria	60418032	635979 ± 131111	500	
P-cyc fungi		216506	2279 ± 607	51
P-cyc bacteria	20928992	220503 ± 45853	51	
N-cyc bacteria	997816	10503 ± 2972	46	

Table S6: Differences in microbial taxonomic diversity (Shannon's H') between grassland and forest sites based on linear mixed models incorporating site pairs, spatial distance, and geological parent material as random factors. The reported p-values are two-tailed.

**Contrast**	*df*	*estimate*	*se*	*t-value*	*p-value*
Fungi					
Managed vs Forest	118	1.37	0.14	2.30	0.021*
Recent vs Forest	142	1.54	0.17	2.53	0.014*
Late vs Forest	145	1.18	0.19	0.37	0.371
Bacteria					
Managed vs Forest	125	1.97	0.08	8.29	0.001***
Recent vs Forest	148	1.66	0.10	4.82	0.001***
Late vs Forest	139	1.65	0.11	4.40	0.001***

Table S7: Differences in microbial taxonomic diversity (Shannon's *H´*) between grasslands in differing successional stages based on linear mixed models incorporating geological parent material as random factor. The reported *p*-values are two-tailed.

**Contrast**	*estimate*	*se*	*z-value*	*p-value*
Fungi				
Managed vs Recent	0.12	0.19	0.61	0.926
Managed vs Late	0.15	0.21	0.69	0.897
Recent vs Late	0.26	0.23	1.13	0.665
Bacteria				
Managed vs Recent	-0.17	0.12	-1.47	0.445
Managed vs Late	0.18	0.13	1.15	0.478
Recent vs Late	0.01	0.14	0.03	1.000

Table S8: Results from permutational multivariate tests (perMANOVA) for differences in community composition between fungal and bacterial OTUs. The reported *p*-values are two-tailed and adjusted for multiple comparisons using Benjamin-Hochberg corrections.

Contrast	*Df*	R^2	*F*	*p-value*
Fungi				
Managed vs Recent	1	0.02	1.29	0.029*
Managed vs Late	1	0.02	1.34	0.017*
Managed vs Forest	1	0.06	5.14	0.001***
Recent vs Late	1	0.03	2.06	0.061
Recent vs Forest	1	0.09	3.98	0.001***
Late vs Forest	1	0.06	2.31	0.001***
Bacteria				
Managed vs Recent	1	0.01	0.92	0.616
Managed vs Late	1	0.02	1.24	0.294
Managed vs Forest	1	0.25	30.3	0.001***
Recent vs Late	1	0.02	0.82	0.704
Recent vs Forest	1	0.20	11.6	0.001***
Late vs Forest	1	0.14	6.47	0.001***

Table S9: Results from two-way ANOVA models testing the effect of forest stand age and evenness on taxonomic and functional gene diversity of fungal and bacterial communities in forest plots.

Diversity variable	***DF***	***F***	***p***	***Effect size***
Fungi				
Taxonomic diversity				
Age	94	1.088	0.299	0.011
Evenness	94	0.268	0.766	0.006
Age:Evenness	94	0.282	0.755	0.006
C-cycling diversity				
Age	41	1.484	0.230	0.035
Evenness	41	0.686	0.509	0.032
Age:Evenness	41	0.039	0.962	0.002
P-cycling diversity				
Age	41	3.400	0.072	0.077
Evenness	41	0.360	0.700	0.017
Age:Evenness	41	0.511	0.604	0.024
Bacteria				
Taxonomic diversity				
Age	99	0.153	0.696	0.002
Evenness	99	0.044	0.957	0.018
Age:Evenness	99	1.547	0.218	0.030
C-cycling diversity				
Age	41	2.272	0.139	0.052
Evenness	41	0.264	0.769	0.013
Age:Evenness	41	0.151	0.860	0.007
P-cycling diversity				
Age	41	1.086	0.303	0.026
Evenness	41	0.357	0.702	0.017
Age:Evenness	41	1.524	0.230	0.069
N-cycling diversity				
Age	41	1.179	0.284	0.028
Evenness	41	1.823	0.174	0.082
Age:Evenness	41	0.336	0.717	0.016

Table S10: Results from permutational analyses of variance (perMANOVA) models testing the effect of forest stand age and evenness on taxonomic and functional gene composition of fungal and bacterial communities in forest plots. The reported *p*-values are two-tailed.

Effect	*SoS*	*R2*	*F*	*p*
Fungi				
Taxonomic composition				
Age	0.443	0.01025	1.0149	0.403
Evenness	1.114	0.0258	1.2844	0.02*
Age:Evenness	2.403	0.05564	1.1077	0.068
C-cycling composition				
Age	0.02212	0.01817	0.8329	0.416
Evenness	0.03114	0.02559	0.5777	0.818
Age:Evenness	0.09284	0.07628	0.6771	0.837
P-cycling composition				
Age	0.01475	0.01496	0.6835	0.729
Evenness	0.03000	0.03044	0.6906	0.839
Age:Evenness	0.08817	0.08944	0.8055	0.795
Bacteria				
Taxonomic composition				
Age	0.1955	0.01183	1.2333	0.216
Evenness	0.2661	0.01611	0.8352	0.598
Age:Evenness	1.0865	0.06578	1.3941	0.063
C-cycling composition				
Age	0.002751	0.02606	1.204	0.284
Evenness	0.003106	0.02942	0.6668	0.721
Age:Evenness	0.010435	0.09883	0.8993	0.566
P-cycling composition				
Age	0.002908	0.0253	1.168	0.274
Evenness	0.003664	0.03187	0.7243	0.672
Age:Evenness	0.012381	0.10771	0.9898	0.432
N-cycling composition				
Age	0.00984	0.01019	0.4634	0.735
Evenness	0.03387	0.03507	0.7996	0.545
Age:Evenness	0.07929	0.0821	0.7334	0.738

Table S11: Differences in microbial genetic diversity (Shannon's *H´*) between grasslands in differing successional stages based on linear mixed models incorporating geological parent material as random factor. The reported *p*-values are two-tailed.

**Contrast**	*estimate*	*se*	*z-value*	*p-value*
Carbon				
Fungi				
Managed vs Recent	-0.08	0.12	-0.64	0.917
Managed vs Late	-0.08	0.12	-0.71	0.890
Recent vs Late	-0.01	0.11	-0.05	0.999
Bacteria				
Managed vs Recent	0.01	0.02	0.12	0.999
Managed vs Late	0.01	0.02	0.57	0.940
Recent vs Late	0.01	0.02	0.69	0.895
Phosphorus				
Managed vs Recent	-0.01	0.01	-0.36	0.983
Managed vs Late	-0.01	0.02	-0.15	0.999
Recent vs Late	-0.01	0.02	-0.49	0.961
Bacteria				
Managed vs Recent	0.01	0.01	0.02	1.000
Managed vs Late	0.01	0.01	0.21	0.997
Recent vs Late	0.01	0.01	0.23	0.995
Nitrogen				
Managed vs Recent	-0.01	0.06	-0.16	0.998
Managed vs Late	-0.05	0.07	-0.71	0.892
Recent vs Late	-0.06	0.06	-0.83	0.809

Table S12: Differences in microbial functional diversity (Shannon's *H´*) between grassland and forest sites based on linear mixed models incorporating site pairs, spatial distance, and geological parent material as random factors. The reported *p*-values are two-tailed.

Contrast	*df*	*estimate*	*se*	*t-value*	*p-value*
Carbon					
Fungi					
Managed vs Forest	90	0.62	0.09	-5.07	0.001***
Recent vs Forest	91	0.61	0.09	-5.39	0.001***
Late vs Forest	75	0.66	0.10	-3.98	0.001***
Bacteria					
Managed vs Forest	68	1.02	0.01	1.14	0.26
Recent vs Forest	68	1.02	0.01	1.44	0.15
Late vs Forest	70	1.00	0.02	0.26	0.79
Phosphorus					
Fungi					
Managed vs Forest	68	0.99	0.01	-1.96	0.05
Recent vs Forest	68	0.99	0.01	-1.87	0.07
Late vs Forest	71	0.99	0.01	-1.74	0.09
Bacteria					
Managed vs Forest	87	1.01	0.02	0.62	0.53
Recent vs Forest	90	1.01	0.02	0.56	0.58
Late vs Forest	58	1.02	0.02	0.88	0.38
Nitrogen					
Bacteria					
Managed vs Forest	68	1.30	0.05	5.17	0.001***
Recent vs Forest	68	1.29	0.05	5.31	0.001***
Late vs Forest	61	1.35	0.05	5.48	0.001***

Table S13: Relationship between taxonomic (Shannon's *H´* of OTU matrices) and functional (Shannon's *H´* of functional matrices) diversity for microbial communities between grassland successional stages, including when divided by ecosystem type (grassland, forest). Results based on ordinary least-square (OLS) regression with two-tailed *p*-values. For bacterial communities, the average genome size (AGS) was included as a covariate in the regressions.

	Df	estimate	se	t-value	p-value	p-value AGS	R^2
Overall community							
Fungal C-cycling	85	-0.15	0.06	-2.58	0.011**	NA	0.07
Fungal P-cycling	85	0.01	0.01	0.53	0.597	NA	0.01
Bacterial C-cycling	90	0.03	0.01	2.86	0.005**	0.001***	0.13
Bacterial P-cycling	90	0.03	0.01	6.29	0.001***	0.001***	0.31
Bacterial N-cycling	90	0.16	0.04	4.17	0.001***	0.901	0.17
Grasslands							
Fungal C-cycling	40	-0.04	0.09	-0.40	0.694	NA	-0.02
Fungal P-cycling	40	0.01	0.01	0.03	0.978	NA	-0.02
Bacterial C-cycling	42	0.02	0.02	0.87	0.392	0.018*	0.10
Bacterial P-cycling	42	0.03	0.01	5.72	0.001***	0.001***	0.41
Bacterial N-cycling	42	-0.04	0.04	-0.97	0.339	0.037*	0.06
Forests							
Fungal C-cycling	43	-0.13	0.06	-2.37	0.022*	NA	0.10
Fungal P-cycling	43	0.01	0.01	0.48	0.631	NA	-0.02
Bacterial C-cycling	44	0.04	0.01	2.99	0.01**	0.012*	0.20
Bacterial P-cycling	44	0.04	0.01	6.55	0.001***	0.002**	0.49
Bacterial N-cycling	44	0.21	0.06	3.61	0.001***	0.456	0.19

Table S14: Results from ordinary least-square regression (OLS) and second-order polynomial (SOP) regression between average niche overlap of C-N-P-cycling genes across the successional gradient. The reported *p*-values are two-tailed.

Process	*estimate*	*se*	*t-value*	*p-value*
C-cycling				
Fungi OLS	-0.01	0.01	-6.66	0.001***
Bacteria OLS	-0.07	0.01	-33.0	0.001***
Bacteria SOP	0.01	0.01	28.2	0.001***
P-cycling				
Fungi OLS	0.04	0.01	4.51	0.001***
Fungi SOP	-0.02	0.01	-8.30	0.001***
Bacteria OLS	-0.01	0.01	-19.3	0.001***
N-cycling				
Bacteria OLS	0.15	0.02	8.09	0.001***
Bacteria SOP	-0.04	0.03	-11.2	0.001***

Table S15: Results from pairwise Wilcoxon Rank-Sum tests for differences in average niche overlap of genes related to nutrient cycling processes between land uses across the successional gradient. *P*-values are two-tailed and have been adjusted using Benjamin-Hochberg correction for multiple testing.

Process	***Statistic***	***p-value***
C-cycling		
Fungi		
Managed vs Recent	227089	0.03*
Managed vs Late	171839	0.001***
Managed vs Forest	521595	0.001***
Recent vs Late	221210	0.331
Recent vs Forest	695984	0.001***
Late vs Forest	661349	0.001***
Bacteria		
Managed vs Recent	140929952	0.001***
Managed vs Late	111815757	0.001***
Managed vs Forest	217382165	0.001***
Recent vs Late	100685397	0.001***
Recent vs Forest	198306258	0.001***
Late vs Forest	163362683	0.001***
P-cycling		
Fungi		
Managed vs Recent	483202	0.071
Managed vs Late	583445	0.001***
Managed vs Forest	1038417	0.001***
Recent vs Late	527436	0.001***
Recent vs Forest	950808	0.001***
Late vs Forest	930685	0.001***
Bacteria		
Managed vs Recent	37474329	0.001***
Managed vs Late	41500356	0.001***
Managed vs Forest	42036157	0.001***
Recent vs Late	37041435	0.286

Recent vs Forest	37301032	0.072
Late vs Forest	40202391	0.338
N-cycling		
Bacteria		
Managed vs Recent	270683	0.462
Managed vs Late	329169	0.974
Managed vs Forest	714627	0.001***
Recent vs Late	256378	0.462
Recent vs Forest	536589	0.001***
Late vs Forest	612059	0.001***

Table S16: Results from pairwise Wilcoxon Rank-Sum tests for differences in average niche overlap of C-cycling genes partitioned across substrate classes. *P*-values are two-tailed and adjusted using Benjamin-Hochberg correction for multiple testing.

Substrate Class	***Contrast***	***n1***	***n2***	***statistic***	***p.adj***
Fungi					
Oligosaccharides	*Managed vs Recent*	48	52	1388	1
Oligosaccharides	*Managed vs Late*	48	50	1452	0.369
Oligosaccharides	*Managed vs Forest*	48	144	4084	0.359
Oligosaccharides	*Recent vs Late*	52	50	1393	1
Oligosaccharides	*Recent vs Forest*	52	144	3923	1
Oligosaccharides	*Late vs Forest*	50	144	3547	1
Cellulose	*Managed vs Recent*	72	78	2860	1
Cellulose	*Managed vs Late*	72	75	2849	1
Cellulose	*Managed vs Forest*	72	240	10926	0.001**
Cellulose	*Recent vs Late*	78	75	3014	1
Cellulose	*Recent vs Forest*	78	240	11380	0.021*
Cellulose	*Late vs Forest*	75	240	10711	0.052
Lignin	*Managed vs Recent*	96	104	5431	0.75
Lignin	*Managed vs Late*	96	100	5257	0.75
Lignin	*Managed vs Forest*	96	192	12970	0.001***
Lignin	*Recent vs Late*	104	100	5178	0.959
Lignin	*Recent vs Forest*	104	192	12670	0.001***
Lignin	*Late vs Forest*	100	192	12126	0.001***
Chitin	*Managed vs Recent*	24	26	340	1
Chitin	*Managed vs Late*	24	25	353	1
Chitin	*Managed vs Forest*	24	144	2256	0.101
Chitin	*Recent vs Late*	26	25	350	1
Chitin	*Recent vs Forest*	26	144	2221	0.655
Chitin	*Late vs Forest*	25	144	2048	1
Other_Polysaccharides	*Managed vs Recent*	48	78	1974	1
Other_Polysaccharides	*Managed vs Late*	48	75	2056	0.74
Other_Polysaccharides	*Managed vs Forest*	48	144	4173	0.19
Other_Polysaccharides	*Recent vs Late*	78	75	3170	1
Other_Polysaccharides	*Recent vs Forest*	78	144	6378	0.478
Other_Polysaccharides	*Late vs Forest*	75	144	5764	1
Mixed	*Managed vs Recent*	24	52	689	0.876
Mixed	*Managed vs Late*	24	50	707	0.876
Mixed	*Managed vs Forest*	24	144	2303	0.055
Mixed	*Recent vs Late*	52	50	1444	0.876
Mixed	*Recent vs Forest*	52	144	4515	0.14
Mixed	*Late vs Forest*	50	144	3992	0.876
Bacteria					
Oligosaccharides	*Managed vs Recent*	590	630	238343	0.001***

Oligosaccharides	*Managed vs Late*	590	565	214566	0.001***
Oligosaccharides	*Managed vs Forest*	590	760	306841	0.001***
Oligosaccharides	*Recent vs Late*	630	565	176395	0.791
Oligosaccharides	*Recent vs Forest*	630	760	257565	0.03*
Oligosaccharides	*Late vs Forest*	565	760	233580	0.018*
Starch/Glycogen	*Managed vs Recent*	354	378	84929	0.001***
Starch/Glycogen	*Managed vs Late*	354	339	75405	0.001***
Starch/Glycogen	*Managed vs Forest*	354	608	151672	0.001***
Starch/Glycogen	*Recent vs Late*	378	339	63307	0.783
Starch/Glycogen	*Recent vs Forest*	378	608	129608	0.01**
Starch/Glycogen	*Late vs Forest*	339	608	117254	0.01**
Peptidoglycan	*Managed vs Recent*	826	882	465349	0.001***
Peptidoglycan	*Managed vs Late*	826	791	397739	0.001***
Peptidoglycan	*Managed vs Forest*	826	1216	654201	0.001***
Peptidoglycan	*Recent vs Late*	882	791	331088	0.144
Peptidoglycan	*Recent vs Forest*	882	1216	551422	0.268
Peptidoglycan	*Late vs Forest*	791	1216	517705	0.011*
Fructan	*Managed vs Recent*	118	126	9422	0.01**
Fructan	*Managed vs Late*	118	113	8684	0.001***
Fructan	*Managed vs Forest*	118	152	12484	0.001***
Fructan	*Recent vs Late*	126	113	7299	0.737
Fructan	*Recent vs Forest*	126	152	10584	0.393
Fructan	*Late vs Forest*	113	152	9303	0.494
Pectin	*Managed vs Recent*	708	756	307259	0.001***
Pectin	*Managed vs Late*	708	565	225237	0.001***
Pectin	*Managed vs Forest*	708	1064	502134	0.001***
Pectin	*Recent vs Late*	756	565	208061	0.422
Pectin	*Recent vs Forest*	756	1064	460161	0.001***
Pectin	*Late vs Forest*	565	1064	353305	0.001***
Xylan	*Managed vs Recent*	118	126	9460	0.01**
Xylan	*Managed vs Late*	118	113	8233	0.01**
Xylan	*Managed vs Forest*	118	304	23430	6.18e-06
Xylan	*Recent vs Late*	126	113	6779	1
Xylan	*Recent vs Forest*	126	304	19937	1
Xylan	*Late vs Forest*	113	304	18688	0.501
Cellulose	*Managed vs Recent*	1180	1134	843738	0.001***
Cellulose	*Managed vs Late*	1180	904	677789	0.001***
Cellulose	*Managed vs Forest*	1180	1824	1434481	0.001***
Cellulose	*Recent vs Late*	1134	904	510525	0.877
Cellulose	*Recent vs Forest*	1134	1824	1098896	0.01**
Cellulose	*Late vs Forest*	904	1824	883788	0.01**
Cellulose/Chitin	*Recent vs Forest*	126	152	9584	0.991
Lignin	*Managed vs Recent*	118	126	8246	0.423

Lignin	*Managed vs Late*	118	113	7292	0.438
Lignin	*Managed vs Forest*	118	304	24191	0.001***
Lignin	*Recent vs Late*	126	113	6964	0.772
Lignin	*Recent vs Forest*	126	304	22368	0.024*
Lignin	*Late vs Forest*	113	304	20562	0.01*
Chitin	*Managed vs Recent*	472	504	145820	0.001***
Chitin	*Managed vs Late*	472	452	131524	0.001***
Chitin	*Managed vs Forest*	472	608	189978	0.001***
Chitin	*Recent vs Late*	504	452	113517	0.928
Chitin	*Recent vs Forest*	504	608	166152	0.034*
Chitin	*Late vs Forest*	452	608	149909	0.034*
Other_Polysaccharides	*Managed vs Recent*	1652	1764	1821480	0.001***
Other_Polysaccharides	*Managed vs Late*	1652	1469	1477844	0.001***
Other_Polysaccharides	*Managed vs Forest*	1652	2432	2711867	0.001***
Other_Polysaccharides	*Recent vs Late*	1764	1469	1244044	0.051
Other_Polysaccharides	*Recent vs Forest*	1764	2432	2332392	0.001***
Other_Polysaccharides	*Late vs Forest*	1469	2432	2021425	0.001***
Mixed	*Managed vs Recent*	236	252	40984	0.001***
Mixed	*Managed vs Late*	236	226	37770	0.001***
Mixed	*Managed vs Forest*	236	456	70612	0.001***
Mixed	*Recent vs Late*	252	226	29294	0.588
Mixed	*Recent vs Forest*	252	456	52560	0.121
Mixed	*Late vs Forest*	226	456	45590	0.043*

Table S17: Results from pairwise Wilcoxon Rank-Sum tests for differences in average niche overlap of P-cycling genes partitioned across pathways. *P*-values are two-tailed and adjusted using Benjamin-Hochberg correction for multiple testing.

Pathway	*Contrast*	*n1*	*n2*	*statistic*	*p.adj*
Fungi					
Organic phosphoester hydrolysis	Managed vs Recent	124	120	7869	0.437
Organic phosphoester hydrolysis	Managed vs Late	124	124	8863	0.113
Organic phosphoester hydrolysis	Managed vs Forest	124	195	15758	0.001***
Organic phosphoester hydrolysis	Recent vs Late	120	124	8280	0.256
Organic phosphoester hydrolysis	Recent vs Forest	120	195	14836	0.001***
Organic phosphoester hydrolysis	Late vs Forest	124	195	14264	0.027*
Oxidative phosphorylation	Managed vs Recent	31	30	497	1
Oxidative phosphorylation	Managed vs Late	31	31	591	0.366
Oxidative phosphorylation	Managed vs Forest	31	39	842	0.028*
Oxidative phosphorylation	Recent vs Late	30	31	511	1
Oxidative phosphorylation	Recent vs Forest	30	39	779	0.092
Oxidative phosphorylation	Late vs Forest	31	39	750	0.346
Pentose phosphate	Managed vs Recent	124	120	7970	0.337
Pentose phosphate	Managed vs Late	124	124	9160	0.028*
Pentose phosphate	Managed vs Forest	124	156	13247	0.001***
Pentose phosphate	Recent vs Late	120	124	8561	0.084
Pentose phosphate	Recent vs Forest	120	156	12388	0.001***
Pentose phosphate	Late vs Forest	124	156	11869	0.01**
Phosphonate and Phospinate metabolism	Managed vs Recent	62	30	966	0.767
Phosphonate and Phospinate metabolism	Managed vs Late	62	62	2405	0.064
Phosphonate and Phospinate metabolism	Managed vs Forest	62	156	6608	0.001***
Phosphonate and Phospinate metabolism	Recent vs Late	30	62	1146	0.145
Phosphonate and Phospinate metabolism	Recent vs Forest	30	156	3121	0.019*
Phosphonate and Phospinate metabolism	Late vs Forest	62	156	5698	0.121
Purine metabolism	Managed vs Recent	403	390	81653	0.341
Purine metabolism	Managed vs Late	403	403	96256	0.001***
Purine metabolism	Managed vs Forest	403	546	149903	0.001***
Purine metabolism	Recent vs Late	390	403	90216	0.001***
Purine metabolism	Recent vs Forest	390	546	142029	0.001***
Purine metabolism	Late vs Forest	403	546	134099	0.001***
Pyrimidine metabolism	Managed vs Recent	186	180	17243	0.619
Pyrimidine metabolism	Managed vs Late	186	217	23786	0.01**
Pyrimidine metabolism	Managed vs Forest	186	312	38524	0.001***
Pyrimidine metabolism	Recent vs Late	180	217	22544	0.016
Pyrimidine metabolism	Recent vs Forest	180	312	36778	0.001***
Pyrimidine metabolism	Late vs Forest	217	312	40356	0.001***
Pyruvate metabolism	Managed vs Recent	62	60	1930	0.918
Pyruvate metabolism	Managed vs Late	62	31	1113	0.651
Pyruvate metabolism	Managed vs Forest	62	156	6348	0.01**
Pyruvate metabolism	Recent vs Late	60	31	1019	0.918
Pyruvate metabolism	Recent vs Forest	60	156	5967	0.01**
Pyruvate metabolism	Late vs Forest	31	156	2855	0.452
Bacteria					
Organic phosphoester hydrolysis	Managed vs Recent	696	720	289929	0.001***
Organic phosphoester hydrolysis	Managed vs Late	696	846	337791	0.001***
Organic phosphoester hydrolysis	Managed vs Forest	696	846	366067	0.001***
Organic phosphoester hydrolysis	Recent vs Late	720	846	300541	0.652
Organic phosphoester hydrolysis	Recent vs Forest	720	846	324142	0.056
Organic phosphoester hydrolysis	Late vs Forest	846	846	386784	0.012*
Others	Managed vs Recent	174	180	18914	0.01**

Others	Managed vs Late	174	282	31016	0.001***
Others	Managed vs Forest	174	282	31069	0.001***
Others	Recent vs Late	180	282	26664	0.882
Others	Recent vs Forest	180	282	26849	0.882
Others	Late vs Forest	282	282	39966	0.916
Oxidative phosphorylation	Managed vs Recent	174	180	18896	0.01**
Oxidative phosphorylation	Managed vs Late	174	188	20409	0.001***
Oxidative phosphorylation	Managed vs Forest	174	188	20402	0.001***
Oxidative phosphorylation	Recent vs Late	180	188	17372	1
Oxidative phosphorylation	Recent vs Forest	180	188	17598	1
Oxidative phosphorylation	Late vs Forest	188	188	18068	1
Pentose phosphate	Managed vs Recent	609	630	228634	0.001***
Pentose phosphate	Managed vs Late	609	658	239621	0.001***
Pentose phosphate	Managed vs Forest	609	658	244892	0.001***
Pentose phosphate	Recent vs Late	630	658	206345	1
Pentose phosphate	Recent vs Forest	630	658	209405	1
Pentose phosphate	Late vs Forest	658	658	222027	1
Phosphonate and Phospinate metabolism	Managed vs Recent	1218	1260	883455	0.001***
Phosphonate and Phospinate metabolism	Managed vs Late	1218	1316	914389	0.001***
Phosphonate and Phospinate metabolism	Managed vs Forest	1218	1316	975208	0.001***
Phosphonate and Phospinate metabolism	Recent vs Late	1260	1316	822509	0.728
Phosphonate and Phospinate metabolism	Recent vs Forest	1260	1316	881036	0.012*
Phosphonate and Phospinate metabolism	Late vs Forest	1316	1316	923772	0.01**
Phosphotransferase	Managed vs Recent	174	180	19047	0.01**
Phosphotransferase	Managed vs Late	174	188	20656	0.001***
Phosphotransferase	Managed vs Forest	174	188	20794	0.001***
Phosphotransferase	Recent vs Late	180	188	17468	1
Phosphotransferase	Recent vs Forest	180	188	17604	1
Phosphotransferase	Late vs Forest	188	188	17610	1
Purine metabolism	Managed vs Recent	1566	1620	1529489	0.001***
Purine metabolism	Managed vs Late	1566	1692	1608826	0.001***
Purine metabolism	Managed vs Forest	1566	1692	1637444	0.001***
Purine metabolism	Recent vs Late	1620	1692	1366181	1
Purine metabolism	Recent vs Forest	1620	1692	1382725	1
Purine metabolism	Late vs Forest	1692	1692	1461560	0.867
Pyrimidine metabolism	Managed vs Recent	957	990	577315	0.001***
Pyrimidine metabolism	Managed vs Late	957	1034	611641	0.001***
Pyrimidine metabolism	Managed vs Forest	957	1034	611464	0.001***
Pyrimidine metabolism	Recent vs Late	990	1034	514984	1
Pyrimidine metabolism	Recent vs Forest	990	1034	511589	1
Pyrimidine metabolism	Late vs Forest	1034	1034	535196	1
Pyruvate metabolism	Managed vs Recent	348	360	77696	0.001***
Pyruvate metabolism	Managed vs Late	348	564	120349	0.001***
Pyruvate metabolism	Managed vs Forest	348	564	121867	0.001***
Pyruvate metabolism	Recent vs Late	360	564	100213	1
Pyruvate metabolism	Recent vs Forest	360	564	99728	1
Pyruvate metabolism	Late vs Forest	564	564	158916	1
Transporters	Managed vs Recent	1218	1530	1125598	0.001***
Transporters	Managed vs Late	1218	1598	1249132	0.001***
Transporters	Managed vs Forest	1218	1598	1186037	0.001***
Transporters	Recent vs Late	1530	1598	1306113	0.01**
Transporters	Recent vs Forest	1530	1598	1219634	0.911
Transporters	Late vs Forest	1598	1598	1176125	0.001***
Two component system	Managed vs Recent	522	540	169979	0.001***
Two component system	Managed vs Late	522	564	177929	0.001***
Two component system	Managed vs Forest	522	564	181157	0.001***
Two component system	Recent vs Late	540	564	151855	1

Two component system	Recent vs Forest	540	564	153684	1
Two component system	Late vs Forest	564	564	161422	1

Table S18: Results from pairwise Wilcoxon Rank-Sum tests for differences in average niche overlap of N-cycling genes partitioned across pathways. *P*-values are two-tailed and adjusted using Benjamin-Hochberg correction for multiple testing.

Pathway	***Contrast***	***n1***	***n2***	***statistic***	***p.adj***
denitrification	Managed vs Recent	174	125	10058	0.72
denitrification	Managed vs Late	174	135	11741	0.996
denitrification	Managed vs Forest	174	204	24625	0.001***
denitrification	Recent vs Late	125	135	9150	0.72
denitrification	Recent vs Forest	125	204	17913	0.001***
denitrification	Late vs Forest	135	204	18858	0.001***
nitrate reduction	Managed vs Recent	319	275	40801	0.284
nitrate reduction	Managed vs Late	319	270	43546	0.815
nitrate reduction	Managed vs Forest	319	408	89711	0.001***
nitrate reduction	Recent vs Late	275	270	40378	0.23
nitrate reduction	Recent vs Forest	275	408	79266	0.001***
nitrate reduction	Late vs Forest	270	408	74760	0.001***
nitrification	Managed vs Recent	232	200	21993	1
nitrification	Managed vs Late	232	216	24895	1
nitrification	Managed vs Forest	232	272	44943	0.001***
nitrification	Recent vs Late	200	216	22298	1
nitrification	Recent vs Forest	200	272	38319	0.001***
nitrification	Late vs Forest	216	272	40819	0.001***
nitrogen fixation	Managed vs Recent	145	50	3992	0.861
nitrogen fixation	Managed vs Late	145	135	9886	0.885
nitrogen fixation	Managed vs Forest	145	306	30865	0.001***
nitrogen fixation	Recent vs Late	50	135	3067	0.861
nitrogen fixation	Recent vs Forest	50	306	9950	0.01**
nitrogen fixation	Late vs Forest	135	306	28500	0.001***

Table S19: Differences in the composition of functional diversity, redundancy, and Simpson's dominance of predicted bacterial metagenomes between successional stages. Results based on permutational multivariate tests (perMANOVA) with Bray-Curtis distances and 10^{4} permutations

Contrast	*Df*	R^2	*F*	*p-value*
Carbon				
Managed vs Recent	1	0.02	1.78	0.140
Managed vs Late	1	0.03	1.75	0.155
Managed vs Forest	1	0.24	47.4	0.001***
Recent vs Late	1	0.01	0.01	0.927
Recent vs Forest	1	0.10	15.2	0.001***
Late vs Forest	1	0.09	12.4	0.001***
Phosphorus				
Managed vs Recent	1	0.03	2.38	0.105
Managed vs Late	1	0.04	2.72	0.078
Managed vs Forest	1	0.46	129	0.001***
Recent vs Late	1	0.01	0.09	0.828
Recent vs Forest	1	0.24	41.2	0.001***
Late vs Forest	1	0.21	33.0	0.001***
Nitrogen				
Managed vs Recent	1	0.09	0.65	0.47
Managed vs Late	1	0.01	0.94	0.382
Managed vs Forest	1	0.40	103	0.001***
Recent vs Late	1	0.01	0.04	0.887
Recent vs Forest	1	0.28	50.2	0.001***
Late vs Forest	1	0.26	45.0	0.001***

Table S20: Effect sizes (*Hedge's g*) of substrate-induced respiration rates between grassland sites at differing stages of succession compared to their respective paired forest sites. Negative effect sizes indicate higher respiration rates in forest sites.

Substrate	***Contrast***	***Hedge's g***	***95% CI***
Glucose	*Managed vs Forest*	-0.44	-0.89 - 0
Glucose	*Recent vs Forest*	-0.5	-1.1 - 0.11
Glucose	*Late vs Forest*	-0.15	-0.79 - 0.49
Glycine	*Managed vs Forest*	-0.66	-1.11 - -0.2
Glycine	*Recent vs Forest*	-0.43	-1.03 - 0.17
Glycine	*Late vs Forest*	-0.26	-0.9 - 0.38
Oxalic Acid	*Managed vs Forest*	-1.18	-1.65 - -0.7
Oxalic Acid	*Recent vs Forest*	-0.85	-1.47 - -0.23
Oxalic Acid	*Late vs Forest*	-0.83	-1.49 - -0.15
Autolyzed yeast	*Managed vs Forest*	-0.69	-1.14 - -0.23
Autolyzed yeast	*Recent vs Forest*	-0.48	-1.08 - 0.13
Autolyzed yeast	*Late vs Forest*	-0.3	-0.94 - 0.34
Lignin	*Managed vs Forest*	-0.66	-1.11 - -0.2
Lignin	*Recent vs Forest*	-0.83	-1.45 - -0.21
Lignin	*Late vs Forest*	-0.5	-1.14 - 0.16
Chitin	*Managed vs Forest*	-0.23	-0.67 - 0.21
Chitin	*Recent vs Forest*	0.45	-0.16 - 1.05
Chitin	*Late vs Forest*	0.00	-0.64 - 0.64
