# Supplementary material for: Functional diversity of soil microbial communities increases with ecosystem development
Source: Nat Commun. 2025 Nov 22;16:10408. doi: 10.1038/s41467-025-66544-8 (PMC12644890; doi:10.1038/s41467-025-66544-8)
Supplement: Supplementary file 2 — Reporting Summary [file 41467_2025_66544_MOESM2_ESM.pdf]

nature portfolio

Corresponding author(s): Tord Ranheim Sveen

Last updated by author(s): Sep 22, 2025

Reporting Summary

Nature Portfolio wishes to improve the reproducibility of the work that we publish. This form provides structure for consistency and transparency in reporting. For further information on Nature Portfolio policies, see our Editorial Policies and the Editorial Policy Checklist.

Statistics

For all statistical analyses, confirm that the following items are present in the figure legend, table legend, main text, or Methods section.

n/a	Confirmed	
☐	☒	The exact sample size (*n*) for each experimental group/condition, given as a discrete number and unit of measurement
☐	☒	A statement on whether measurements were taken from distinct samples or whether the same sample was measured repeatedly
☐	☒	The statistical test(s) used AND whether they are one- or two-sided<br>*Only common tests should be described solely by name; describe more complex techniques in the Methods section.*
☐	☒	A description of all covariates tested
☐	☒	A description of any assumptions or corrections, such as tests of normality and adjustment for multiple comparisons
☐	☒	A full description of the statistical parameters including central tendency (e.g. means) or other basic estimates (e.g. regression coefficient) AND variation (e.g. standard deviation) or associated estimates of uncertainty (e.g. confidence intervals)
☐	☒	For null hypothesis testing, the test statistic (e.g. *F*, *t*, *r*) with confidence intervals, effect sizes, degrees of freedom and *P* value noted<br>*Give P values as exact values whenever suitable.*
☒	☐	For Bayesian analysis, information on the choice of priors and Markov chain Monte Carlo settings
☒	☐	For hierarchical and complex designs, identification of the appropriate level for tests and full reporting of outcomes
☐	☒	Estimates of effect sizes (e.g. Cohen's *d*, Pearson's *r*), indicating how they were calculated

Our web collection on statistics for biologists contains articles on many of the points above.

Software and code

Policy information about availability of computer code

Data collection	The pipeline to process metabarcoding samples is available under https://lotus2.earlham.ac.uk/main.php?site=downloads (https://github.com/hildebra/lotus2). The pipeline to process shotgun metagenomic samples is available under https://github.com/hildebra/MATAFILER (https://doi.org/10.5281/zenodo.5831723). The carbohydrate active enzymes (CAZy) Database is available under http://www.cazy.org/, the nitrogen cycling gene (NCyc) Database is available under https://github.com/qichao1984/NCyc (https:doi.org/10.1093/bioinformatics/bty741), the phosphorus cycling gene (PCyCDB) Database is available under https://github.com/ZengJiaxiong/Phosphorus-cycling-database<br>(https://doi.org/10.1186/s40168-022-01292-1). The Evolutionary genealogy of genes: Non-supervised Orthologous Groups (EggNOG) Database is available under http://eggnog5.embl.de/#/app/home. The Kyoto Encyclopedia of Genes and Genomes (KEGG) Database is available under https://www.genome.jp/kegg/kegg1.html.<br>Plant functional traits gathered from the TRY database, ver. 6.0 (available under: https://www.try-db.org/TryWeb/Home.php)
Data analysis	R (ver. 4.4.1) used to build custom code for data analyses, with statistical analyses performed using the following packages and version<br>effectsize (1.0.1)<br>vegan (2.7.1)<br>adiv (2.2.1)<br>lme4 (1.1.37)<br>MicroNiche (1.0.0)<br>rfPermute (2.5.5)<br>SRS (0.2.3)

glmm.hp (0.1.8)

MATAFILER (ver. 2.1) was used to process shotgun metagenome reads
LOTUS2 (ver. 2.3.4.1) was used to process amplicon reads
Picrust2 (ver. 2.5.2) was used to predict bacterial metagenomes

For manuscripts utilizing custom algorithms or software that are central to the research but not yet described in published literature, software must be made available to editors and reviewers. We strongly encourage code deposition in a community repository (e.g. GitHub). See the Nature Portfolio guidelines for submitting code & software for further information.

Data

Policy information about availability of data

All manuscripts must include a data availability statement. This statement should provide the following information, where applicable:

- Accession codes, unique identifiers, or web links for publicly available datasets
- A description of any restrictions on data availability
- For clinical datasets or third party data, please ensure that the statement adheres to our policy

The carbohydrate active enzymes (CAZy) Database is available under http://www.cazy.org/, the nitrogen cycling gene (NCyc) Database is available under https://github.com/qichao1984/NCyc [https:doi.org/10.1093/bioinformatics/bty741], the phosphorus cycling gene (PCyCDB) Database is available under https://github.com/ZengJiaxiong/Phosphorus-cycling-database
[https://doi.org/10.1186/s40168-022-01292-1]. The Evolutionary genealogy of genes: Non-supervised Orthologous Groups (EggNOG) Database is available under http://eggnog5.embl.de/#/app/home. The Kyoto Encyclopedia of Genes and Genomes (KEGG) Database is available under https://www.genome.jp/kegg/kegg1.html. The data supporting the results and figures in this study are provided in the Supplementary Information/Source Data file.
Picrust2 (ver. 2.5.2) was used to predict bacterial metagenomes and is available under (https://huttenhower.sph.harvard.edu/picrust/)

Data Availability
The 16S and ITS metabarcoding data generated in this study have been deposited in the NCBI Sequence Read Archive (SRA) under the Bio Project accession number: PRJNA994701 [https://www.ncbi.nlm.nih.gov/bioproject/PRJNA994701/]. All soil metagenomic sequences and associated metadata have been deposited in the Sequence Read Archive (SRA) under accession number: PRJEB56463 [https://www.ncbi.nlm.nih.gov/sra/?term=PRJEB56463].
Soure data is available at the project's GitHub repository: https://github.com/tranheim/Succession_functional_diversity/tree/main

Code Availability
Codes to reproduce analyses and figures are accessible at the project's GitHub repository. accessible at: https://github.com/tranheim/Succession_functional_diversity/tree/main

Research involving human participants, their data, or biological material

Policy information about studies with human participants or human data. See also policy information about sex, gender (identity/presentation), and sexual orientation and race, ethnicity and racism.

Reporting on sex and gender	N/A
Reporting on race, ethnicity, or other socially relevant groupings	N/A
Population characteristics	N/A
Recruitment	N/A
Ethics oversight	N/A

Note that full information on the approval of the study protocol must also be provided in the manuscript.

Field-specific reporting

Please select the one below that is the best fit for your research. If you are not sure, read the appropriate sections before making your selection.

☐ Life sciences ☐ Behavioural & social sciences ☒ Ecological, evolutionary & environmental sciences

For a reference copy of the document with all sections, see nature.com/documents/nr-reporting-summary-flat.pdf

Ecological, evolutionary & environmental sciences study design

All studies must disclose on these points even when the disclosure is negative.

Study description	This study utilizes high-throughput sequencing methods for metabarcoding and shotgun metagenomics of soil bacterial and fungal communities and their functional genes, soil physiochemical measurements, and experimentally measured rates of substrate-induced respiration to study the effects of land abandonment and vegetation succession on the structure and function of soil microbial communities undergoing land-use change. The study was conducted across a latitudinal graident of 1600 km spanning the whole of Sweden. A total of 207 sites were selected (105 forest, 102 grassland) out of which 92% were paired grassland forest sites based on a geographical proximity criterion (< 6.5 km between paired sites, median distance = 3.63 km). The grassland sites were further divided into managed (n = 49), recently abandoned (n = 30), and late-stage successional (n =23) based on vegetation characteristics, for a total of four treatment groups together with forests (n = 105). Substrate-induced respiration was measured on soil collected from a subset of 156 sites, all grassland-forest pairs.
Research sample	Each research sample was a composite of 15 top soil samples (3 x 10 cm), except experiment samples for substrate-induced respiration (SIR) which were 5g dry-weight equivalent subsamples in the lab. In each soil sample we isolated DNA to amplify bacterial and fungal communities using PCR and amplicon sequencing. Shotgun metagenomic sequencing was performed on a subset (n = 95) of DNA samples from paired grassland-forest sites.
Sampling strategy	Sampling locations were part of Sweden's environmental monitoring programs. A total of 207 sampling locations across Sweden were selected based on management/abandonment status (grasslands) and proximity to grasslands (forests). Sampling was undertaken between July-September 2020 by TRS (lead author). No statistical test was used to determine sampling size. Instead, we determined this to satisfy the following criteria: Geographical extent, covering the whole of Sweden to accurately reflect ongoing scale of land abandonment; proximity between grassland and forest samples to minimize environmental variation between sample pairs; and sufficient coverage in each treatment category based on minimum feasible sample size for statistical analysis from prior experience of the authors e.g. Bahram et al. https://nph.onlinelibrary.wiley.com/doi/full/10.1111/nph.16598
Data collection	Data was collected from soil samples and experiment (SIR). Molecular lab work was undertaken by the first author and by the Mycology lab at SLU Uppsala, amplicon and shotgun metagenomic sequencing was performed on Illumina NovaSeq 6000 using 250 bp paired end reads by a commercial lab, soil nutrients were measured at a commercial lab, soil C and N was performed by the fifth and sixth authors, SIR experiment was performed by the first and fourth author.
Timing and spatial scale	Soil sampling was undertaken once per site between the 2nd of July until the 12th of September 2020 covering the three major climate zones of Sweden and spanning a total area of ~400 000 km2
Data exclusions	Three bacterial samples were discarded due to poor sequencing depth (< 3000 reads) and 8 fungal samples were discarded due to poor sequencing depth (< 300 reads). For the shotgun metagenome sequencing and SIR experiments, a susbet of samples (shotgun sequencing, n = 95; SIRn = 156) out of the total 207 samples were selected due to high expenses. These subsamples were selected based on the following two criteria: geographic cover across all major biomes of Sweden; and paired grassland-forest sites
Reproducibility	Each soil sample was pooled from several subsamples (n = 15), two successful technical PCR, replicates were pooled per sample before metabarcoding sequencing.
Randomization	Both grassland and forest sites are included in environmental monitoring programs where initial grid placement across Sweden's entire terrestrial area are randomized prior to inventorying and classification.<br>More information about the grassland environmental monitoring program is available at https://www.slu.se/institutioner/ekologi/foma1/jordbruk/regional-landskapsovervakning/ (Swedish only) and information about Sweden's national forest inventory program can be found at https://www.slu.se/en/Collaborative-Centres-and-Projects/the-swedish-national-forest-inventory/
Blinding	Blinding was not performed as field sampling was undertaken in natural field settings where expertise was required to infer successional stage (grassland) and tree composition (forests) based on plant communities.

Did the study involve field work? ☒ Yes ☐ No

Field work, collection and transport

Field conditions	Sampling was done under variable weather conditions which did not influence the work. Temperature and rainfall were obtained for the soil samples from the climate database and did not influence sampling time nor location. For detailed description, see Methods.
Location	Soil samples were taken from 207 locations across Sweden (Fig. 1) and for site distribution across treatment groups see Supplementary Table 1
Access & import/export	Soil samples were collected from locations in public domain or in previous agreement with the local community and/or property owner.
Disturbance	The field work caused minimal environmental disturbance when collecting soil samples

Reporting for specific materials, systems and methods

We require information from authors about some types of materials, experimental systems and methods used in many studies. Here, indicate whether each material, system or method listed is relevant to your study. If you are not sure if a list item applies to your research, read the appropriate section before selecting a response.

Materials & experimental systems

n/a	Involved in the study	
☒	☐	Antibodies
☒	☐	Eukaryotic cell lines
☒	☐	Palaeontology and archaeology
☒	☐	Animals and other organisms
☒	☐	Clinical data
☒	☐	Dual use research of concern
☐	☒	Plants

Methods

n/a	Involved in the study	
☒	☐	ChIP-seq
☒	☐	Flow cytometry
☒	☐	MRI-based neuroimaging

Dual use research of concern

Policy information about dual use research of concern

Hazards

Could the accidental, deliberate or reckless misuse of agents or technologies generated in the work, or the application of information presented in the manuscript, pose a threat to:

No	Yes	
☒	☐	Public health
☒	☐	National security
☒	☐	Crops and/or livestock
☒	☐	Ecosystems
☒	☐	Any other significant area

Experiments of concern

Does the work involve any of these experiments of concern:

No	Yes	
☒	☐	Demonstrate how to render a vaccine ineffective
☒	☐	Confer resistance to therapeutically useful antibiotics or antiviral agents
☒	☐	Enhance the virulence of a pathogen or render a nonpathogen virulent
☒	☐	Increase transmissibility of a pathogen
☒	☐	Alter the host range of a pathogen
☒	☐	Enable evasion of diagnostic/detection modalities
☒	☐	Enable the weaponization of a biological agent or toxin
☒	☐	Any other potentially harmful combination of experiments and agents

Plants

Seed stocks	N/A
Novel plant genotypes	N/A
Authentication	N/A
